# Estimating the economic impact of gender-based violence on women survivors: A comparative study of support program interventions in Makueni and Naivasha, Kenya

**DOI:** 10.1016/j.aprim.2023.102840

**Published:** 2023-12-22

**Authors:** Grace Wamue-Ngare, Pacificah Okemwa, Isaac Kimunio, Okumba Miruka, Grace Okong’o, Pauline Kamau, Lucy Maina, Jane Njuguna, Lilian Kiruja, Simon Okoth

**Affiliations:** a Kenyatta University, Kenya; b Independent Consultant, Kenya; c University of Nairobi, Kenya; d Tharaka Nithi County Government, Kenya

**Keywords:** Gender-based violence, Economic impact, Health risks, Survivors, Prevention programs, Violencia de género, Impacto económico, Riesgos de salud, Supervivientes, Programas de prevención

## Abstract

**Objective:**

To estimate the economic cost of GBV from the perspective of a women survivor who sought help from two identified programs (Makueni GBVRC and Life Bloom Services International [LBSI]).

**Design:**

A mixed method research design combining qualitative and quantitative approaches.

**Site:**

Makueni GBVRC in Makueni County, overseen by the Makueni County government, and LBSI in Naivasha, Nakuru County, a non-profit organization devoted to serving local communities.

**Participants:**

Study participants include women survivors of GBV, aged 18 and above, actively seeking services at Makueni GBVRC and LBSI.

**Interventions:**

The study adopts a qualitative approach to delve into the intricate economic costs of GBV on survivors. Additionally, quantitative data analysis employs an accounting model to ascertain the financial implications.

**Main measurements:**

The costs analyses were done from the perspective of the women survivors. An accounting model was utilized to evaluate the cost of GBV on selected survivors. Furthermore, the research explores the enduring consequences for survivors, including psychological trauma and susceptibility to stress-related diseases.

**Results:**

The findings reveal substantial economic costs linked to GBV, adversely affecting survivors, their children, and society at large. These costs encompass direct expenditures on medical care, legal representation, and counseling, as well as indirect costs, such as lost productivity.

**Conclusions:**

Beyond immediate and indirect costs, the study underscores the existence of opportunity costs—what survivors and affected children could attain in the absence of GBV.

## Abstract (es)

**Objetivo:**

Estimar el costo económico de la violencia de género desde la perspectiva de una mujer sobreviviente que buscó ayuda de dos programas identificados (Makueni GBVRC y Life Bloom Services International [LBSI]).

**Diseño:**

Un diseño de investigación de método mixto que combina enfoques cualitativos y cuantitativos.

**Sitio:**

Makueni GBVRC en el condado de Makueni, supervisado por el gobierno del condado de Makueni, y LBSI en Naivasha, condado de Nakuru, una organización sin fines de lucro dedicada a servir a las comunidades locales.

**Participantes:**

Los participantes del estudio incluyen mujeres sobrevivientes de violencia de género, de 18 años o más, que buscan activamente servicios en Makueni GBVRC y LBSI.

**Intervenciones:**

El estudio adopta un enfoque cualitativo para profundizar en los intrincados costos económicos de la violencia de género para los sobrevivientes. Además, el análisis de datos cuantitativos emplea un modelo contable para determinar las implicaciones financieras.

**Mediciones principales:**

Los análisis de costos se realizaron desde la perspectiva de las mujeres sobrevivientes. Se utilizó un modelo contable para evaluar el costo de la violencia de género en sobrevivientes seleccionados. Además, la investigación explora las consecuencias duraderas para los supervivientes, incluido el trauma psicológico y la susceptibilidad a enfermedades relacionadas con el estrés.

**Resultados:**

Los hallazgos revelan costos económicos sustanciales relacionados con la violencia de género, que afectan negativamente a los sobrevivientes, sus hijos y la sociedad en general. Estos costos abarcan gastos directos en atención médica, representación legal y asesoramiento, así como costos indirectos, como la pérdida de productividad.

**Conclusiones:**

Más allá de los costos inmediatos e indirectos, el estudio subraya la existencia de costos de oportunidad: lo que los sobrevivientes y los niños afectados podrían lograr en ausencia de violencia de género.

## Introduction

The prevalence of gender based violence (GBV) in Kenya remains high with data showing that 35% of women and girls are affected.^1^ The adverse outcomes are extreme for survivors but spread to families and economies in a variety of ways. Studies emphasize psychological and health effects and hardly considers economic costs and losses that are often long-term and at times irreversible. A 2018 World Bank report estimates the value of economic loss due to GBV at about 1.2%–3.7% in developing countries’ gross domestic product (GDP). Globally, however, UN Women puts it at 2% of the global GDP.^2^

The effects of GBV can be immediate, such as missing to report to paid or unpaid work, mental and physical malady, poor access to services, and loss of time and property.^3^, ^4^, ^5^ Medium and long-term effects include: stagnated professional development, education and career mobility; disability; morbidity; domestic instability; and loss of quality life.^6^ In its extremities, GBV causes loss of life and untold suffering for dependents, especially women and girls who are disproportionately affected due to gender disparities. Gender-based violence (GBV) places a substantial economic burden on women survivors and the wider community, extending to the national level. Kenya's economy experiences a significant loss of GDP ranging between 7.8% and 10% due to gender-based violence (GBV).^1^

In Kenya, the healthcare system is often the first point of contact for survivors of GBV seeking help. Services for GBV survivors are in most cases provided free of charge.^7^ However, the extent of primary care's involvement in GBV can be influenced by various factors including policy, training, resources, and cultural attitudes. The “GBVRC Kenya” refers to Gender-Based Violence Recovery Centers in Kenya, which are pivotal in aiding survivors of gender-based violence. These centers adopt a comprehensive care model for sexual violence survivors, providing an array of services that include medical care, counseling, legal aid, and economic empowerment programs. In Kenya, those affected by abuse can find refuge and assistance at GBVRCs. The efforts of GBVRCs in Kenya are often supported by non-governmental organizations (NGOs), such as Life Bloom Services International (LBSI). These NGOs play a critical role in augmenting the services provided by GBVRCs, which may include additional support such as community outreach, advocacy, training, and resources that might not be readily available through government-funded programs.

Although there is some evidence on general national losses attributed to GBV, there remains a gap as to the indirect actual loss of incomes to families, survivors and employers, as well as productive time for businesses. This study explored some forms of hidden and unhidden costs of GBV in Kenya. Demonstrating these costs through evidence-based data makes a case for the public, private and informal sectors that manage GBV to shift focus to investing in prevention programs, at the same time strengthening government policy and synchronizing response actions. The evidence may be utilized to enhance advocacy work at family, community, government, private and economic sectors to increase investments in GBV prevention programs and protect survivors from the vice.

### Problem statement

Anecdotal evidence in Kenya reveals that GBV has a substantial economic impact on women and girls as well as communities.^7^ Though estimations of economic expenditure on GBV in Kenya exist, the focus has been on cost of services provided to survivors. This is often centered on selected health services delivery points. There is thus need for systematic analysis of the immediate and long-term costs of GBV on survivors. Furthermore, there is little focus on accounting for losses due to GBV, which often require a multi-dimensional approach and use of robust statistical testing. In addition, some costs relating to GBV remain unexplored and invisible due to the difficulty in accounting for them since they take a longtime to manifest. Studies have rarely looked at financial costs of GBV at individual and cumulative levels. Additionally, there is paucity of studies estimating the monetary value of losses incurred by women as indirect costs. Data to link the effect of GBV on survivors’ recovery and their economic empowerment is equally limited.

Primary Health Care (PHC), with its frontline role in addressing the immediate health needs of communities, finds itself in a pivotal position when it comes to managing GBV aftermath. The World Health Organization (WHO), recognizing the integral role of PHC in the global health framework, underscores its significance concerning GBV.^8^ By exploring the economic implications of GBV in Kenya, this study hopes to shed light on the immense challenges encountered by Primary Health Care centers. Understanding these challenges within their economic context offers insights into the necessary resource allocations, training, and upgrades required in PHC facilities. By aligning our findings with WHO's recommendations, we aim to offer a holistic view of the problem, underscoring the urgency of informed interventions.

## Materials and methods

### Research design

This study adopted a mixed method research design combining qualitative and quantitative approaches to conduct an exploration of the economic cost of GBV on survivors. Specifically, an accounting model was utilized to evaluate the cost of GBV on selected survivors. This model multiplies the unit cost of a service by the number of times the service was provided to each beneficiary.^5^ The total cost of GBV is then simply the sum of all the distinct categories of costs. In addition, a Burden of Disease’ design was used to affirm the indirect costs, which spreads over some period of time to a woman survivor. The burden of disease is the impact of a health problem on a given population as a consequence of GBV. It can be measured using a variety of indicators, such as work-time lost due to GBV-related illness, the associated reduction in labor productivity, and the financial cost of dealing with a GBV incidence, such as an expense on legal or counseling services. The burden of disease (BOD) approach, introduced in 1993, has been suggested as a valuable tool for health planning and priority setting, utilizing metrics like disability adjusted life years (DALYs).^9^, ^10^ A qualitative approach to this methodology was chosen for this study due lack of good data on morbidity, mortality, age of onset, disability severity, and societal preferences for future health and years of healthy life at various ages that is used to calculate DALYs. This is comprehensively quantified with the excess disease burden attributable to exposure to GBV as a risk factor for disease and injury. By so doing, health risks and effects of GBV on survivors was investigated.

### Study sites

The research was conducted among survivors who had sought assistance at two distinct locations: Makueni GBVRC in Makueni County and Life Bloom Services International (LBSI) in Naivasha, Nakuru County. Makueni GBVRC, operated by the Makueni County government, is a specialized facility dedicated to addressing and preventing GBV. It offers a range of support services for GBV survivors, including medical care, legal aid, psychosocial support, and other forms of assistance. The primary objective of Makueni GBVRC is to aid survivors in their recovery and empowerment while promoting gender equality within the community. On the other hand, LBSI is a non-profit organization based in Naivasha, Kenya, with a mission to improve the lives of local communities by providing essential services such as education, healthcare, and clean water. LBSI also extends support and resources to individuals and families affected by poverty, HIV/AIDS, and GBV.

### Sampling procedure

A complete enumeration survey method was employed, aiming to include every eligible participant in the study. This approach is ethically sound and reliable for GBV research, aligning with Reliefweb's (2018) recommendations for conducting research on GBV in communities.^11^ The distribution of respondents across the two facilities was 61% from Makueni GBVRC and 39% from LBSI-Naivasha.

### Data collection research instruments/tools

Data collection involved the use of an interview schedule and a structured questionnaire administered to women survivors who had sought services from the two service providers. The recruitment of participants adhered to the ethical and safety guidelines outlined by the World Health Organization for research on violence against women.^12^

### Inclusion and exclusion criteria

To be included in the study, women survivors had to meet specific criteria, which included being 18 years or older, having sought services from RRRP/GBVRC, and expressing a willingness to participate. The research scope did not encompass costs incurred by society due to GBV-related deaths.

### Data analysis

An interpretative analysis was done on qualitative data thus aiding in understanding the indirect costs of the abuse suffered by women.

Quantitative data was analyzed using an accounting model to establish the economic costs of GBV. The costs were calculated based on the experiences of the survivors the last one year after seeking services at the programs. The costs analyses were done from the perspective of the women survivors. The following is the study flow chart.



**Study flow chart**

The study used the following formulas for calculating indirect costs:

#### Lost working days

The study used the following Equation 1 to determine the GBV cost as a result of lost working hours

(1) $$\text{COWDL}=\Sigma \{FE_{i}\times FD_{i}\}$$

where COWDL – cost of working days lost, *FE*_*i*_ – daily earning per woman, *FD*_*i*_ – lost days from work.

#### Cost of lost school days

The study determined the cost of lost school days for children, which have implication on mothers using Eq. (2) below.

(2) $$\text{COSDL}=\Sigma \{C_{i}\times LD_{i}\}n$$

where COSDL – cost of school days lost, *C*_*i*_ – daily school fees cost, *LD*_*i*_ – days lost by the woman's children, and *n* – number of children affected.

The annual school fees for the different school levels were estimated from secondary data sources. Secondary school level was taken as Ksh. 40,000 while University and college level fees were taken as Ksh. 40,000.^13^, ^14^, ^15^ Even though primary education is free in Kenya, parents experience some costs such as for meals, books and uniforms. Some parents also prefer to take their children to private schools. For the pre-primary and primary school levels, fees were taken as Ksh. 14,000.^13^, ^14^, ^15^ The number of school days in a year was taken to be 180.^16^ The daily school fees were derived by dividing the annual school fees by the number of school days in a year.

#### Lost domestic work

To estimate the cost of domestic work, the maximum number of working hours per day was set at 10, with a monetary value calculated by the assumption that a paid domestic house worker gets an average daily wage^17^ of Ksh. 300 [USD 3]. Eq. (3) below was used to estimate this variable.

(3) $$\text{CODWDL}=\Sigma_{j}\Sigma_{i}\;\{RW_{i}\times AH_{i}XD_{i}\}$$

where CODWDL – cost of domestic work days lost, *RW*_*i*_ – minimum hourly wage rate in household service for women, *AH*_*i*_ – daily hours spent in domestic work by the woman/survivor, *D*_*i*_ – woman number of days lost for domestic work.

#### Total indirect costs

(4) TIDC=COWDL+COSDL+CODWDL

#### Total cost

Total GBV costs were calculated by a summation of direct and indirect costs using the formula below.

(5) TGBVC=TDOPC+TIDC

where TGBVC is total GBV costs; TDOPC is total direct out of pocket costs; and TIDC is total indirect costs.

### Study objective

The objectives of the study was to:

Estimate the economic cost of GBV from the perspective of a women survivor who sought help from two identified programs (Makueni GBVRC and Life Bloom Services International in Nakuru).

Asses the health impacts of GBV among women survivors in Makueni Gender Based Violence Recovery Center and Life Bloom Services International in Nakuru.

## Results

### Demographic characteristics of survivors

This section presents demographic data on respondents by age, level of education, marital status as shown in Table 1.

**Table 1:** Demographic characteristics.

Variable	Indicator	Makueni GBVRC		LBSI (Naivasha)	
		Frequency	Percentage	Frequency	Percentage
Age	18–25 years	16	17.6	5	8.6
	26–35 years	28	30.8	27	46.6
	36–45 years	26	28.6	18	31
	46–55 years	16	17.6	5	8.6
	Above 55 years	5	5.5	3	5.2
	Total	91	100	58	100

Level of education	Never attended school	2	2.2	9	15.5
	Primary	34	37.4	18	31
	Incomplete primary	4	4.4	12	20.4
	Secondary	30	33	9	15.5
	Incomplete Secondary	1	1.1	2	3.4
	College	9	11.7	5	9.2
	University	2	2.2	1	1.7
	Total	91	100	58	100

While data in Table 2 shows that GBV cuts across all the age groups considered, majority (79%) out of the 149 participants were within the 26–45 cohort while only 19.5% were between 18 and 25 years as well as above 55. This means that GBV normally occurs at the economic productive age of an individual denying one a chance to actively participate in economic activities. This findings are in line with the Kenya Demographic and Health Survey (KDHS)^18^ of 2022 which reported that thirty-four percent of women in Kenya have experienced physical violence since age 15.

**Table 2:** Direct GBV costs.

Cost items	Makueni GBVRC	LBSI (Naivasha)
	Estimated cost per survivor (Ksh)	Estimated cost per survivor (Ksh)
Cost of lost/damaged property	17,500 [USD 175]	17,000 [USD 170]
Medical costs	7344 [USD 73.44	5069 [USD 50.69]
Transport costs	3635 [USD 36.35]	1389 [USD 13.89]
Legal costs	6286 [USD 62.86]	2583 [USD 25.83]
Counseling costs	3921 [USD 39.21]	3000 [USD 30]
Accommodation costs	5000 [USD 50]	5692 [USD 56.92]
Costs as a result of your children missing school due to the violence	33,965 [USD 339.65]	5400 [USD 54]
Other costs	500 [USD 5]	49,402 [USD 494]
Total	71,865 [USD 718.65]	89,535 [USD 895.35]

### Economic costs of GBV

Direct costs are actual expenditures incurred by women survivors while indirect costs represent the value of lost labor productivity due to GBV. The total cost is simply the sum of all the distinct categories of costs. These costs are presented both in Kenyan Shillings (Ksh) and their equivalent value in US Dollars (USD). The exchange rate used is Kshs 100 to 1 USD.

#### Direct costs

Direct costs refer to actual cash-paid expenses calculated in monetary terms. They include: costs of lost/damaged property, cost for medical care, transport, legal services, counseling, accommodation, and those experienced as a result of children missing school due to the violence. Analyzed separately for Makueni GBVRC and LBSI, respectively, the estimates depended primarily on data collected from the women survivors. The results are shown in Table 2.

Table 2 provides a detailed breakdown of the estimated costs incurred by the survivors. Survivors at Makueni GBVRC reported an average cost of Ksh 17,500 [USD 175] for lost or damaged property, Ksh 7344 [USD 73.44] for medical expenses, Ksh 3635 [USD 36.35] for transportation, Ksh 6286 [USD 62.86] for legal costs, Ksh 3921 [USD 39.21] for counseling, Ksh 5000 [USD 50] for accommodation, and Ksh 33,965 [USD 339.65] for children missing school due to violence. Additionally, there were unspecified “other costs” averaging Ksh 500 [USD 5]. In total, the average direct cost for Makueni GBVRC survivors was Ksh 71,865 [USD 718.65]. Comparatively, survivors from LBSI (Naivasha) had slightly lower costs for most categories but reported a higher average total cost of Ksh 89,535 [USD 895.35].

#### Indirect costs

The study went further to compute the indirect costs, which were measured in terms of productivity losses whose monetary values could be estimated. Variables used were lost working days for survivors and lost school days for children.

##### Lost working days

From the survey data, the average monthly income for women survivors in Makueni GBVRC was Ksh 5188 [USD 51.88], translating to a daily income of Ksh 172.93 [USD 1.7293]. 27 working days were lost in GBV cost for the woman survivor due to lost working days. Therefore, the average cost of working days lost per woman survivor in Makueni GBVRC was 4669 [USD 46.69].

The average monthly income for women survivors in LBSI (Naivasha) was Ksh 8913 [USD 89.13] translating to a daily income of Ksh 297.1 [USD 2.971]. As a result of the violence, the woman survivor incurred GBV costs of 29 lost working days. Consequently, the average cost of working days lost per woman survivor in LBSI (Naivasha) was Ksh. 8616 [USD 86.16].

##### Cost of lost school days

Survivors with children indicated that the violence they faced trickled down to their children, especially in terms of missing school as the mothers sought alterative accommodation and health care. The text below exemplifies some of the problems experienced by children of survivors.

• changing schools
• Looking for new schools
• Acquiring new school uniform as children left all their clothes, books and uniforms behind as they fled,
• Purchase of new stationary,
• Medical treatment for children injured, psychological counseling fees, legal services, shelter and accommodation provision.
• Failing to attend school as a resulted sexual abuse.
• Being thrown out of the homes by the abusive fathers.

The costs as a result of one's children missing school due to violence reveal varying financial demands. For school enrollment, costs are higher in Naivasha (Ksh 10,545 or USD 105.45) compared to Makueni (Ksh 7333 or USD 73.33), indicating potentially pricier schools or additional enrollment fees. Uniform and stationery expenses also differ, with Naivasha's Ksh 4899 [USD 48.99] surpassing Makueni's Ksh 3500 [USD 35], suggesting a local price variation for these items.

Medical treatment costs are quite similar, with Makueni slightly higher at Ksh 6333 [USD 63.33] versus Naivasha's Ksh 6000 [USD 60], encompassing potential doctor's fees and medications. In contrast, psychosocial counseling shows a substantial discrepancy, with Makueni at Ksh 9333 [USD 93.33] and Naivasha at a mere Ksh 500 [USD 5], hinting at differences in service delivery or subsidization.

Shelter costs, covering accommodation like temporary housing, are higher in Naivasha (Ksh 5333 or USD 53.33) than in Makueni (Ksh 3533 or USD 35.33), which may reflect the cost of housing or the extent of services provided. ‘Other costs’—a category including transportation, legal fees, and food—stand out with Naivasha's considerable Ksh 22,125 [USD 221.25] dwarfing Makueni's Ksh 3933 [USD 39.33], implying a broader service range or additional survivor needs.

The cumulative cost per survivor totals to Ksh 49,402 [USD 494.02] for Naivasha and Ksh 33,965 [USD 339.65] for Makueni. The stark overall difference suggests Naivasha's higher living costs, pricier services, or a more extensive support network.

Table 3 shows the cost of lost school days. The total cost was calculated by multiplying the duration in days the affected child(ren) missed school by the daily school fees cost.

**Table 3:** Cost of lost school days.

School level	Number of children affected	Annual school fees cost (Ksh)	Daily cost (Ksh)	Duration in days the affected child(ren) missed school	Total cost (Ksh)
*Makueni GBVRC*					
Pre-primary	2	14,000	78	81	6300
Primary	20	14,000	78	466	36,244
Secondary	12	40,000	222	400	88,889
University	1	24,000	133	60	8000
College	1	24,000	133	60	8000
Total cost					147,433 [USD 1474.33]
Number of women survivors affected					31
Average cost					4756 [USD 47.56]

School level	Number of children	Annual cost (Ksh)	Daily cost (Ksh)	Duration in days the affected child(ren) missed school	Total cost (Ksh)
*LBSI (Naivasha)*					
Pre-primary	10	14,000	78	228	17,733
Primary	20	14,000	78	389	30,256
Secondary	2	40,000	222	64	14,222
University	0	24,000	133	0	–
College	1	24,000	133	60	8000
Total cost					70,211 [USD 702.11]
Number of women survivors affected					26
Average cost					2700 [USD 27]

From Table 3, the total cost of lost school days in Makueni was Ksh. 147,433 [USD 1474.33]. The number of women survivors whose children missed school was 31. This results in an average cost of lost school days per woman in Makueni at Ksh. 4756 [USD 47.56].

The total cost of lost school days in LBSI was Ksh. 70,211 [USD 702.11]. Using the sample of 26, the average cost of lost school days was Ksh. 2700 [USD 27] per woman survivor.

##### Lost domestic work

The study established that on average, 27 domestic work days were lost by women in Makueni, with 10 h spent on domestic work per day and an average hourly wage^8^ is Ksh. 30 [USD 0.3]. This gives an average of Ksh. 8100 [USD 81] per month lost in terms of domestic work per woman survivor in Makueni.

From the Naivasha sample, an average of 29 domestic work days was lost. Assuming 10 hours are spent on domestic work per day and the average hourly wage^17^ for domestic work is Kshs. 30 [USD 0.3]. This gives an average of Ksh. 8700 [USD 87] lost in terms of domestic work per woman survivor in Naivasha.

##### Total indirect costs

The total indirect costs of GBV were analyzed and the results presented separately for the two study sites. The results for Makueni GBVRC show that the average total indirect costs per woman were Ksh. 17,525 [USD 175.25]. This comprised of: average cost of lost working days per woman at Ksh. 4669 [USD 46.69], average cost of lost school days per child at Ksh. 4756 [USD 47.56] and average cost of lost domestic work at Ksh. 8100 [USD 81].

The results for LBSI show that the average total indirect cost per woman was Ksh. 20,016 [USD 200.16]. This was higher than that of Makueni GBVRC. The findings show that in LBSI, lost working days by the woman survivor was Ksh. 8616 [USD 86.16]. The value of lost domestic work was Ksh. 8700 [USD 87] while lost school days for children amounted to Ksh. 2700 [USD 27].

#### Total cost

Lastly, the study computed the total GBV cost in the two regions. The results show that the average direct cost per woman survivor was higher than the indirect costs in both regions at Ksh. 161,400 [USD 1614]. The total GBV cost realized by the survivors was Ksh. 109,551 [USD 1095.51] in LBSI and Ksh. 89,390 [USD 893.9] in Makueni. These data imply that GBV has heavy economic costs often borne by survivors. Although such costs are incurred by individual survivors, they also have an impact on the national economy since a lot of funds are used to address issues that could be avoided, if GBV was absent. These huge amounts of money could be channeled to other development activities that would improve the standards of living and welfare of citizens.

### Non-monetary costs: health risks of GBV

Having looked at the cost of SGBV on survivors, the study went further to investigate long term effects after its occurrence. The study delved into non-monetary costs associated with the health risks of gender-based violence (GBV) to highlight the concealed expenses beyond the direct and indirect financial burdens. These non-monetary costs shed light on the broader and often overlooked impacts of GBV on survivors and society.

Besides incurring huge economic costs as shown above, the GBV often leave survivors with scars, some which never heal. Nevertheless, these often predispose one to numerous stress-related diseases; which otherwise could be avoided, if one was not exposed to incidences of violence. As such, the study sought to estimate the health risks of GBV among women in Makueni and Naivasha using the burden of disease qualitative explanation.

Table 4 is a comparative analysis of the consequences of GBV for respondents in Makueni GBVRC and LBSI.

**Table 4:** Comparative analysis of GBV consequences for the two study sites.

Facility	Condition	Frequency	%
Makueni GBVRC	Anger	5	5.5
	Anxiety	1	1.1
	Constant fear	2	2.2
	Depression	13	14.3
	Emotional disturbance	35	38.5
	Feelings of hopelessness	1	1.1
	Permanent disability	2	2.2
	Physical injury	27	29.7
	Sexual dysfunction	3	3.3
	Suicide	1	1.1
	Total	91	100

LBSI (Nakuru)	Anger	1	1.7
	Constant fear	1	1.7
	Emotional disturbance	7	12.1
	Loss of self-esteem	1	1.7
	Others	1	1.7
	Physical injury	37	63.8
	Recurrent pain or discomfort	6	10.3
	Sexual dysfunction	1	1.7
	Sexual transmitted infections	3	5.2
	Total	58	100

*Source*: Study data (March, 2022).

The analysis above shows that approximately 38.5% of survivors in Makueni were emotionally disturbed compared to 12.1% in LBSI. Up to 63.8% in LBSI experienced physical injuries with the corresponding figure in Makueni being 29.7%. Constant fear was more prominent in Makueni than LBSI. This could be related to the fact that the survivors in Makueni were largely staying with their partners while most of those in LBSI may not have been.

The results in Table 5 show the distribution of GBV consequences by age across the two study sites.

**Table 5:** Percentage distribution of GBV consequences by age.

Health outcomes	Age group (years)				
	18–25	26–35	36–45	46–55	Above 55
*Makueni GBVRC*					
Suicide			4		
Constant fear			4	6	
Physical injuries	25	39	31	19	20
Depression	18	18	8	25	
Anxiety		4			
Emotional disturbance	38	32	35	50	60
Sexual dysfunctions			13		
Permanent disability	6				20
Anger	13	7	4		
Total	100	100	100	100	100

*LBSI-Naivasha*					
Recurrent pain/discomfort	20	11	10		
Constant fear			6		
Physical injuries	40	70	55	60	100
Emotional disturbance	20	7	17	40	
Sexually transmitted infections			6		
Sexual dysfunctions	20	8			
Anger			6		
Loss of self-esteem		4			
Total	100	100	100	100	100

*Source*: Study data (March, 2022).

In Makueni, the results show that physical injuries and emotional disturbance were prevalent in virtually all age groups. For LBSI, recurrent pain/discomfort featured strongly. Overall, the results show that GBV has varied health implications on survivors, ranging from physical to the sexual, psychological and economic. These consequences have an adverse effect on women's economic productivity and quality of life. To eliminate them, it is imperative to prevent GBV.

The verbatim below are from the women of survivors.

- “*I was constantly under stress and under fear of violence and violence itself. Especially after my hand was broken and I was unable to work for 3 months*.”
- “*Uterus removed as it was damaged so this is a loss more than monetary*.”
- “*I got emotionally disturbed and could not trust men*.”
- “*I could not get out of the house due to shame from the neighbours*.”
- “*Chronic forgetfulness, I can plan to work on something then I forget.*”

## Discussion

The total direct and indirect costs of GBV constitute a profound economic detriment, diverting resources that could otherwise be invested in productive economic activities. Costs related to healthcare, transportation, legal representation, and counseling not only deplete individual resources but also affect entire families, contributing to increased poverty. The profound financial implications of gender-based violence (GBV) on survivors are unambiguous. The direct costs for a woman survivor (combining both regions) averaged higher than the indirect costs at Ksh. 161,400 [USD 1614]. There is a significant economic burden of GBV, affecting not just individual survivors, but also reflecting implications on the broader national economy. Despite primary education being free in Kenya, there are still associated costs that parents bear, impacting their financial capability. There is a clear difference in economic impact between Makueni GBVRC and LBSI, especially in terms of lost working days and lost school days, which needs strategic interventions tailored for each area. The findings corroborate with other studies that there is a huge economic loss due to GBV.^5^, ^7^, ^19^, ^20^, ^21^, ^22^

GBV against women has huge economic costs that affect survivors, as well as their children leave alone the entire society. The findings corroborate other studies, which noted that children exposed to GBV are negatively affected as they end up having low self-esteem, a factor that compromises their rights.^23^, ^24^ It can have severe and long-lasting impacts on human health. These findings are supported by other similar studies.^25^, ^26^, ^27^ Besides high costs to society in terms of time loss and resources, increased use of health services and facilities divert already scarce resources thereby exacerbating an already existing disease burden. This is a drawback to the health sector, which has an impact on the economy. If GBV was adequately addressed through meaningful prevention measures, costs incurred addressing it would be saved, and health facilities would be free to counter the disease burden.

Beyond direct expenses, GBV has opportunity costs, reflecting what women survivors could achieve if they didn’t face the consequences of violence. This includes advancements in careers, community contributions, and overall economic progress. Children affected by GBV may also miss out on their full potential due to disrupted education and psychological trauma. Although there is some evidence on general national losses attributed to GBV, there remains a gap as to the indirect actual loss of incomes to families, survivors and employers, as well as productive time for businesses. Further, unlike NGEC (2016), which was a country study, the current study's focus was on survivors who had sought services at Makueni GBVRC and Life Bloom Services International (LBSI) in Naivasha, Nakuru County. Demonstrating these costs through evidence-based data makes a case for the public, private and informal sectors that manage GBV to shift focus to investing in prevention programs, at the same time strengthening government policy and synchronizing response actions. The evidence may be utilized to enhance advocacy work at family, community, government, private and economic sectors to increase investments in GBV prevention programs and protect survivors from the vice. While the study focused on specific locations, Makueni GBVRC and LBSI in Nakuru, its findings suggest that GBV's economic impact could be representative of a larger regional or national challenge. When scaled to a national level, the economic consequences of GBV become staggering, hindering Kenya's economic growth and development.

Given GBV's significant economic impact, there is a compelling case for increased investment in GBV prevention programs. Allocating resources to prevention measures, such as public awareness campaigns, legal reforms, and support networks, can lead to long-term cost savings.: Economic empowerment of women is also a preventive measure that can yield direct and indirect benefits. Ensuring women have economic independence and access to resources can help them break free from abusive situations and drive societal shifts in gender roles and norms, ultimately reducing the prevalence of GBV.

A study by CARE reveals that survivors bear the biggest cost reflected in social, emotional and economic realms but also that states and not-for-profit entities bear significant costs in provision of services while the private sector incurs losses in terms of working hours. However, this study explored economic costs of GBV in Kenya from the perspective of a woman survivor who sought help from two identified programs (Makueni GBVRC and LBSI). In-depth face to face interviews were conducted with the women GBV survivors to gain a deeper understanding of their experiences in the RRRPs. These interviews ensured that participants gave answers in their own words. The interviews aimed to record own feelings, impressions, and changes experienced by the GBV survivors.

The study, however, had some limitations:

• The study was constrained by a limited sample size, due to the nature of the problem. However, we made an effort to interview each and every participant who sought GBV help from the two study sites. Costs of GBV are far reaching and distinct.
• The study does not account for the costs associated with victims who did not survive. Such losses encompass not just the loss of these individuals’ contributions to the economy but also the opportunity costs related to the resources spent on treatment that ultimately proved unsuccessful. The research focused on survivors, thereby excluding mortality-related data and concentrating on women who were alive during the study period.
• The costs documented may not reflect the full extent of GBV's economic impact, as the data were collected solely from those who sought help from the specified centers. Those with serious injuries or disability might have sought help from better equipped facilities such as from the City. However, the findings might point toward the invisible nature of many GBV injuries, which might include psychological and emotional.

Despite the limitations, this study makes a substantial contribution by exploring the long-term effects of sexual and gender-based violence (SGBV) beyond immediate costs. It uncovers hidden non-monetary costs associated with the health risks of gender-based violence (GBV), providing a comprehensive understanding of its broader societal impacts. Additionally, the study highlights that apart from substantial economic costs, GBV leaves enduring scars on survivors, increasing their susceptibility to stress-related diseases.

## Conclusion

The study has shown that gender-based violence (GBV) has a significant impact on the health of survivors, including physical, sexual, psychological and economic consequences. These consequences not only affect the survivors’ economic productivity and quality of life, but also have an adverse effect on their children and society as a whole. It is therefore crucial to prevent GBV in order to eliminate these negative effects. The study also highlights the high economic costs of GBV on survivors, their children and society, including lost productivity and increased use of health services. Addressing GBV through prevention measures would not only save these costs, but also free up resources in the health sector to address other disease burdens.

In conclusion, GBV is not merely a social issue; it carries substantial economic implications affecting individuals, communities, and nations. Understanding the comprehensive economic costs of GBV highlights the urgency for preventive measures and interventions. Addressing GBV promotes social justice and serves as a prudent economic strategy for nations striving for sustainable development.

## Data availability statement

Data available on request.

## Ethical considerations

As standard procedure, the research proposal was submitted to Kenyatta University Ethics Review Committee (KUERC) for evaluation to ascertain that the research approaches adhered to the standards set for such type of studies.

All researchers and enumerators were required to understand, sign and adhere to a Code of Conduct. All were trained on how to create and maintain a safe environment for GBV survivors and take measures to prevent, detect and appropriately respond to any likelihood of abuse or exploitation. Detailed information about possible risks of participation in the study was included in the informed consent form including sensitive types of questions that were therein. This enhanced informed decision making for participation.

To ensure anonymity of respondents, codes were used as much as possible to minimize need for personal identifiable information on data collection tools and generated raw data. Targeted respondents were assured that their names and personal details would be anonymized in the report and identifiers would be immediately removed after analysis. The research also made a commitment to have the raw data collected, recorded verbatim and field notes destroyed three years after completion of the activity.

Meetings with targeted respondents to explain the study and get consent were held in private, secure and discreet locations in order not to raise curiosity. Targeted survivors were asked to indicate a place of preference where they could meet the researcher(s) for the initial meetings to ensure that they were not exposed to the public and their safety and security not jeopardized. Privacy and discreetness was maintained at all times to avoid stigmatization on the part of the survivors.

Participants were informed of their right to withdraw from the study at any point without any implications on their part. The researchers continuously sought confirmation from respondents that consent to participate in the study was still valid.

## Funding

We acknowledge funding from the Bill and Melinda Gates Foundation. Kenyatta University received a Research Grant from Bill & Melinda Gates Foundation (Ref: INV-002289) to establish a hub whose purpose is to generate a body of evidence to advance Women's Economic Empowerment (WEE) in Kenya. This research was one the studies funded by the grant.

## Conflict of interest

No potential conflict of interest was reported by the authors.
