# Supplementary material for: Estimating the economic impact of gender-based violence on women survivors: A comparative study of support program interventions in Makueni and Naivasha, Kenya
Source: Aten Primaria. 2023 Dec 22;56(10):102840. doi: 10.1016/j.aprim.2023.102840 (PMC11662271; doi:10.1016/j.aprim.2023.102840)
Supplement: Supplementary file 1 [file mmc1.doc]

**Tool A - Women Survivors-22**

| Question | Answer |
| --- | --- |
| Name of Enumerator | <1> Dr. Isaac Kimunio  <2> Prof. Ngare  <3> Dr.Okemwa  <4> Prof. Maina  <5> Dr. Okong’o  <6> Dr. Wanjira  <7> Dr. Kamau  <8> Dr. Miruka  <9> Lilian Kiruja  <10> Simon Okoth  <11> Baron Musyoka  <12> Julia Wambui  <13> Meriba Mwende  <14> Peninah Ndonye  <15> Stella Muasya  <16> Laura Wanjohi |
| Sex of Enumerator | <1> Male  <2> Female |
| Date of survey |  |
| Hold start Time |  |
| My name is {0} I am a Researcher at Kenyatta University conducting an evaluation study entitled “Impact of programmes and Initiatives addressing Sexual and Gender-Based Violence as a Constraint to Women’s Economic Empowerment”. The information will be used to improve response to and prevention of SGBV in Kenya hence enable more women to transit to economic empowerment beyond recovery. |  |
| Respondent Code |  |
| Name of facility | <1> Life Bloom Services International  <2> Makueni GBVRC  <3> Other (specify) |
| Type of facility | <1> Shelter  <2> GBVRC  <3> Programme  <4> Other (specify) |
| County where facility is located | <1> Nairobi  <2> Nakuru  <3> Makueni  <4> Tharaka Nithi |
| Physical location of the facility | <1> Urban  <2> Rural |
| **SECTION 1: DEMOGRAPHIC CHARACTERISTICS.** |  |
| 1.1. Gender of the respondent | <1> Male  <2> Female  <3> Other |
| 1.2. Age | <1> 18- 25 years  <2> 26-35 years  <3> 36-45 years  <4> 46-55 years  <5> Above 55 years |
| 1.3. Religion/denomination | <1> Christian - Catholic  <2> Christian - Protestant  <3> Christian - Pentecostal/Evangelical  <4> Muslim  <5> Africanist  <6> Hindu  <7> No Response  <8> Other (Specify) |
| Q1.4a. What is your current place of residence? | <1> Gilgil  <2> Kabati  <3> Karagita  <4> Kayole  <5> KCC  <6> Kihoto  <7> Lakeview  <8> Naivasha  <9> Police Line  <10> Sanctuary  <11> Site  <12> Wote  <13> Kathozweni  <14> Mukuyuni  <15> Kalawa  <16> Kalamba  <17> Other (Specify) |
| 1.4b. Did you ever change your residence due to the SGBV you experienced? | <1> Yes  <2> No |
| 1.4c. If YES, where were you staying before the violence? | <1> Rural area  <2> Urban – low-income estate  <3> Urban – middle-income estate  <4> Urban - upper-income estate |
| 1.5. What was the size of your previous place of residence? | <1> 1 room  <2> 2 rooms  <3> One bedroomed house  <4> Two bedroomed house  <5> Three bedroomed house  <6> Other (Explain) |
| 1.6. Who owned your previous place of residence? | <1> Rented  <2> Self  <3> Jointly owned with partner/husband  <4> Other (Explain) |
| 1.7. What is the size of your current place of residence? | <1> 1 room  <2> 2 rooms  <3> One bedroomed house  <4> Two bedroomed house  <5> Three bedroomed house  <6> Other (Explain) |
| 1.8. Who owns your current place of residence? | <1> Rented  <2> Self  <3> Jointly owned with partner/husband  <4> Other (Explain) |
| 1.9. How long have you lived in your current place of residence? | <1> Less than a year  <2> 1 - 2 years  <3> 3 - 4 years  <4> 5 - 6 years  <5> Over 6 years |
| 1.10. Whom do you live with? | <1> No one  <2> My children  <3> My male friend  <4> My female friend  <5> My siblings  <6> My spouse  <7> My parents  <8> Other (specify)……… |
| Q1.11. Highest level of formal education attained | <1> None  <2> Primary school certificate  <3> Secondary school certificate  <4> College certificate  <5> College diploma  <6> Bachelor’s degree  <7> Master’s degree  <8> PhD  <9> No response  <10> Other (please specify) |
| 1.12a. Do you have any other training apart from formal education? | <1> Yes  <2> No |
| 1.12b. If yes, please specify the skills you have acquired through the training. |  |
| 1.13. Please indicate your marital status before the violence occurred. | <1> Single  <2> Married  <3> Living with a partner  <4> Separated  <5> Divorced |
| 1.14. How old were you when you got married /began to live with a partner? | <1> Not applicable  <2> Below 18 years  <3> 18- 25 years  <4> 26-35 years  <5> 36-45 years  <6> 46-55 years  <7> Above 55 years  <8> Can't remember |
| 1.15. Current marital status | <1> Single  <2> Married  <3> Widowed  <4> Separated  <5> Divorced  <6> Cohabiting  <7> No response |
| 1.16. How long have you stayed with your current/recent partner? | <1> Not applicable  <2> Less than a year  <3> 1-2 years  <4> 3-4 years  <5> 5-6 years  <6> 7+ years  <7> Not sure/Don't know |
| 1.17a. Did your husband/partner (perpetrator of the violence) have any other partners while being married to or having a relationship with you? | <1> Yes  <2> No  <3> Don't know  <4> N/A |
| 1.17b. If response to 1.17a is “Yes”, explain how or whether those relationships contributed to the violence experienced |  |
| q1.18. a. Who was the perpetrator of the violence? | <1> Boyfriend (Current/former)  <2> Husband (Current/former)  <3> Other marital family member (brother-in-law, sister-in-law, mother-in-law, father in law)  <4> Blood relative (brother, sister, father, mother, uncle)  <5> Stranger  <6> Service provider (specify in the space below)  <7> Neighbour  <8> Employees  <9> Employer (current/former)  <10> Workmate/colleague/fellow student  <11> Other (specify)  <12> Other (specify) |
| Q1.18. b. What was the highest formal educational qualification of the perpetrator? | <1> None  <2> Primary school certificate  <3> Secondary school certificate  <4> College certificate  <5> College diploma  <6> Bachelor’s degree  <7> Master’s degree  <8> PhD  <9> No response  <10> Other (please specify) |
| Q1.18c. Age of the perpetrator at the time of the violence | <1> Below 18 years  <2> 18- 25 years  <3> 26-35 years  <4> 36-45 years  <5> 46-55 years  <6> Above 55 years  <7> I don’t know |
| Q1.18d. Religion of the perpetrator at the time of the violence | <1> Christian - Catholic  <2> Christian - Protestant  <3> Christian - Pentecostal/Evangelical  <4> Muslim  <5> Africanist  <6> Hindu  <7> No Response  <8> Other (Specify) |
| **SECTION 2: EMPLOYMENT AND INCOME.** |  |
| Q2.1. Which of the following best describes the work you do? | <1> Unpaid family worker/housewife  <2> Salaried employment  <3> Self-employment (non-agricultural)  <4> Self-employment (agricultural)  <5> Casual work (daily piece work)  <6> Short term Contract worker  <7> Others (specify) |
| Q2.2. What is your main source of income? | <1> Salaried employment  <2> Self-employment (non-agricultural)  <3> Self-employment (agricultural)  <4> Casual work (daily piece work)  <5> Short term contract work  <6> Other (specify) |
| Q2.3. How many hours per day do you spend in productive (paid) work? | <1> 1 - 2  <2> 3 - 4  <3> 5 - 6  <4> 7 - 8  <5> Over 8  <6> I don’t know |
| Q2.4. What was your income (per month) before joining the program/RRRP/GBVRC? | <1> Nil  <2> Ksh. 10,000 and below  <3> Ksh. 10,001 – 20,000  <4> Ksh. 20,001 - 30,000  <5> Ksh. 30,001 - 40,000  <6> Ksh. 40,001 - 50,000  <7> More than Kshs 50,000 |
| Q2.5. Who controlled (decided on how to spend) the money you earned before the program? | <1> Myself  <2> My husband/partner at own will  <3> My husband and I  <4> My husband/partner against own will  <5> Other (Explain) |
| Q2.6. What is your current income (per month) after the program / RRRP/GBVRC? | <1> Nil  <2> Ksh. 10,000 and below  <3> Ksh. 10,001 – 20,000  <4> Ksh. 20,001 - 30,000  <5> Ksh. 30,001 - 40,000  <6> Ksh. 40,001 - 50,000  <7> More than Kshs 50,000 |
| Q2.7. Who controls (decides on how to spend) the money you earn after going through the program? | <1> Myself  <2> My husband/partner at own will  <3> My husband and I  <4> My husband/partner against own will  <5> Other (Explain) |
| Q2.8 (a). Did your income improve after going through the program? | <1> Yes  <2> No  <3> Not applicable |
| Q2.8(b). Explain why you say {0} in the previous question |  |
| **SECTION 3: HELP FROM RRRPs/GBVRCs.** |  |
| 3.1a. Please indicate the type of violence you experienced? | <1> Sexual (e.g. forced to have sexual intercourse, unnatural sexual acts -anal/oral sex etc)  <2> Physical (e.g. slapping, hitting, kicking, throwing objects that can hurt, shoving etc)  <3> Social (barred from visiting parents/relatives/friends, attending social gatherings etc)  <4> Economic (stopped from working, dispossessed of money, denied opportunity to do business)  <5> Psychological/emotional (insults, humiliation, intimidation, threats)  <6> Cultural (e.g. FGM, forced inheritance)  <7> Other (Explain) …  <8> Not applicable |
| Q3.1b. How many times, on average, were you violated in a month before you took action? | <1> Below 5  <2> 5-10  <3> 11- 15  <4> 16- 20  <5> Over 20  <6> I lost count |
| Q3.1c. Where did the violence occur? | <1> At home  <2> At work  <3> Both at home and at work  <4> Other (specify) |
| Q3.1 d. On which day of the week was the violence mainly perpetrated? | <1> Mondays  <2> Tuesdays  <3> Wednesdays  <4> Thursdays  <5> Fridays  <6> Saturdays  <7> Sundays  <8> All days |
| Q3.1e. At what time of the day was the violence mostly perpetrated? | <1> Morning  <2> Afternoon  <3> Evening  <4> At night  <5> All times  <6> No specific time |
| Q3.1 f. What would you say triggered the violence in most cases? | <1> Money  <2> Alcohol  <3> Food  <4> Partner’s extra-marital affairs  <5> Accusations that I had extra-marital affairs  <6> Cultural factors  <7> Other (specify) |
| Q3.1g. Did you ever separate with the perpetrator because of the violence? | <1> Yes  <2> No  <3> Not applicable |
| Q3.1h. If yes, did you go back to your partner after the program? | <1> Yes  <2> No |
| Q3.1j. If yes, please explain what made you go back. |  |
| Q3.2 a) Did you ever report the perpetrator to a third party? | <1> Yes  <2> No |
| Q3.2 b) If yes, who did you report to? | <1> My parents  <2> His parents  <3> My relatives  <4> His relatives  <5> My friends  <6> His friends  <7> Elders  <8> Police  <9> Local administration (e.g. Chief…..)  <10> Neighbours  <11> Religious leaders  <12> Others – please explain |
| Q3.2c. What actions were taken against the perpetrator by the person/people you reported to? | <1> None  <2> Perpetrator could not be traced (went into hiding)  <3> Perpetrator was arrested and charged in court  <4> Perpetrator was arrested but released  <5> Perpetrator was warned  <6> A family meeting was held to resolve the matter  <7> Others (Explain) |
| Q3.3. For how many years did you withstand the abuse before seeking help? | <1> Less than 2  <2> 2  <3> 3- 5  <4> 6-8  <5> 9-11  <6> 12- 14  <7> More than 15  <8> Not applicable |
| Q3.4. Please describe the point at which you felt that you could not cope with further violence and decided to seek help? |  |
| Q 3.5. Who did you seek help from? | <1> No one  <2> My parents  <3> His parents  <4> My relatives  <5> His relatives  <6> My friends  <7> His friends  <8> Elders  <9> Police  <10> Local administration (e.g. Chief…..)  <11> Neighbours  <12> Religious leaders  <13> GBVRC/RRRP  <14> Hospital  <15> Others (Specify) |
| Q 3.6 a) What kind of help did you seek? |  |
| Q 3.6b. Did you receive the help you sought? | <1> Yes  <2> No |
| Q 3.7. Did the violence compel you to do any of the following | <1> Desert the house/home  <2> Report to the police  <3> Report to his/my parents  <4> Report to my/his relatives  <5> Report to local administrators  <6> Report to religious leaders  <7> Report to elders  <8> Other (specify)1  <9> Other (specify)2 |
| Q 3.8. How did the person/people you reported to react? | <1> Ignored the report  <2> Blamed me  <3> Convened a meeting for both of us  <4> Summoned both of us for counselling  <5> Supported the perpetrator  <6> Reported to local administrators  <7> Advised me to leave the partner/husband  <8> Advised me to return to the partner/husband  <9> Advised me to go to a shelter, GBVRC or hospital  <10> Other (Explain) |
| 3.9. Did the incident compel you to seek any of the following services? |  |
| *[Q3_9_1] Legal* | *<1> Yes*  *<2> No* |
| *[Q3_9_2] Medical* | *<1> Yes*  *<2> No* |
| *[Q3_9_3] Psychological (counselling)* | *<1> Yes*  *<2> No* |
| *[Q3_9_4] Shelter/RRRP* | *<1> Yes*  *<2> No* |
| *[Q3_9_5] Other (specify in the space or enter 'none' if no)* | *<1> Yes*  *<2> No* |
| Q3.10a. Please indicate the kind of help you were given by the RRRP/GBVRC/shelter? | <1> Medical treatment  <2> Psychological support (counselling)  <3> Legal information/referral  <4> Court evidence  <5> Employment  <6> Rescue/shelter/housing  <7> Formal education  <8> Start-up loan  <9> Business training  <10> Child support  <11> Other (specify) |
| Q 3.10 b. For how long, on average, did you receive the services from the shelter/GBVCR/RRRP? | <1> Less than a month  <2> 1-3 months  <3> 4-6 months  <4> 7 – 12 months  <5> More than a year |
| Q 3.10c. Which year did you join the program shelter/GBVCR/RRRP? | <99> Can't remember |
| **SECTION 4: COST OF SGBV.** |  |
| Q 4.1. How much time in total did you spend in accessing the services from the GBVCR/RRRP/shelter? | <1> 1-7 days  <2> A month  <3> More than one month  <4> Still on going  <5> Other (specify) |
| Q4.2.a. Did you have to stop house work due to the violence? | <1> Yes  <2> No  <3> Not applicable |
| Q 4.2b. Please explain your answer above |  |
| Q 4.3.a. Did you have to take time off your daily routine (work) due to the violence? | <1> Yes  <2> No  <3> Not applicable |
| Q 4.3b. If yes, how much time did you take off? | <1> Less than one week  <2> 1 week  <3> Two weeks  <4> Three weeks  <5> Four weeks  <6> More than one month |
| Q 4.3c). Explain why you needed that time off your daily routine. |  |
| Q 4.3d). If no, explain why you didn't take time off your daily routine due to the violence? |  |
| Q4.3e. Kindly estimate how much income you lost per day from time taken off your daily routine (state the amount). | <1> None  <2> Ksh. 10,000 and below  <3> Ksh. 10,001 – 20,000  <4> Ksh. 20,001 - 30,000  <5> Ksh. 30,001 - 40,000  <6> Ksh. 40,001 - 50,000  <7> More than Kshs 50,000  <8> Cannot remember |
| Q4.4a. Did the perpetrator take time off from his daily routine due to the violence? | <1> Yes  <2> No  <3> Does not apply  <4> I don't know |
| Q 4.4b. If the perpetrator was husband/partner how much was he earning during the period of violence? | <1> Nil  <2> Ksh. 10,000 and below  <3> Ksh. 10,001 – 20,000  <4> Ksh. 20,001 - 30,000  <5> Ksh. 30,001 - 40,000  <6> Ksh. 40,001 - 50,000  <7> More than Kshs 50,000  <8> I don’t know |
| Q 4.5a. Were any of your children also violated? | <1> Yes  <2> No  <3> Does not apply |
| Q 4.5b. If yes to question 4.5.a, please explain nature of violation |  |
| Q4.5c. Did any of your children have to miss school due to the violence? | <1> Yes  <2> No  <3> Not applicable |
| Q4.5d. If yes to question 4.5c, what level of schooling are the affected children in? | <1> Pre-primary  <2> Primary  <3> Secondary  <4> College  <5> University |
| Q4.5e. For what duration did the affected child(ren) miss school? | <1> Less than one week  <2> 1 week  <3> Two weeks  <4> Three weeks  <5> Four weeks  <6> More than a month |
| Q 4.5f. Did you incur any costs as a result of your children missing school due to the violence? | <1> Yes  <2> No  <3> Not applicable |
| Q4.5g. If yes, specify what you incurred the costs on | <1> New school  <2> School uniform and stationery  <3> Medical treatment  <4> Psychosocial counselling  <5> Legal services  <6> Shelter/accommodation  <7> Other (explain) |
| 4.5h. If yes to the above, estimate the amount you spent per item |  |
| *[Q4_5h_1] New school* |  |
| *[Q4_5h_2] School uniform and stationery* |  |
| *[Q4_5h_3] Medical treatment* |  |
| *[Q4_5h_4] Psychosocial counselling* |  |
| *[Q4_5h_5] Legal services* |  |
| *[Q4_5h_6] Shelter/accommodation* |  |
| *[Q4_5h_7] {0}* |  |
| Q4.6a. Did you/the child(ren) lose any property during the incident (e.g. destruction of clothes, loss of money, loss of phone, jewellery, etc.)? | <1> Yes  <2> No  <3> Does not apply |
| Q4.6(b). If yes, estimate the monetary value of the property lost | <1> Nil  <2> Ksh. 10,000 and below  <3> Ksh. 10,001 – 20,000  <4> Ksh. 20,001 - 30,000  <5> Ksh. 30,001 - 40,000  <6> Ksh. 40,001 - 50,000  <7> Kshs 50,000- 100,000  <8> Kshs 100,001- 500,000  <9> More than Kshs 500,000  <10> Any other amount (please indicate)  <11> Don't know  <12> Can't remember |
| Q4.7. To what extent did the violence you experienced affect your economic situation? | <1> Not at all  <2> Little extent  <3> Great extent  <4> Don't know |
| Q4.8a. Did you suffer any other form of loss that is not possible to express in monetary terms? | <1> Yes  <2> No  <3> Not applicable |
| Q4.8b. If yes, please explain |  |
| Q4.9. Did you suffer any of the following as a result of the violence? | <1> Physical injury  <2> Permanent disability  <3> Recurrent pain or discomfort  <4> Sexual dysfunctions  <5> Premature birth  <6> Sexually transmitted infections  <7> Emotional disturbance  <8> Constant fear  <9> Anger  <10> Depression  <11> Anxiety  <12> General hatred for men  <13> Thoughts of suicide  <14> Feelings of hopelessness  <15> Breakdown in marital or romantic relationships  <16> Loss of self-esteem  <17> Loss of ability to work and fend for self/family  <18> Diversion of resources to treatment  <19> Dependence on others  <20> Loss of income  <21> Loss of respect in the community or neighbourhood  <22> Smoking  <23> Drug abuse  <24> Alcohol abuse  <25> Other (Explain) |
| Q4.10a. Would you say that the incident affected your ability to perform your daily work (usual formal work, household duties, study, family and social activities)? | <1> Yes  <2> No  <3> Not applicable |
| Q4.10b. If yes, please elaborate |  |
| Q4.11. Did you incur any expenses on services related to recovery from the violence? | <1> Yes  <2> No  <3> Not applicable |
| 4.12. If yes, estimate how much you spent on the following? |  |
| *[Q4_12_1] Transport to access the services* | *<1> Not applicable*  *<2> Below Ksh. 1000*  *<3> Ksh. 1000 - 5000*  *<4> Ksh. 5001 - 10,000*  *<5> Ksh. 10,001 - 15,000*  *<6> Ksh. 15,001 - 20,000*  *<7> Above Ksh. 20,001*  *<8> Don't know*  *<9> Can't remember* |
| *[Q4_12_2] Payment for medical services* | *<1> Not applicable*  *<2> Below Ksh. 1000*  *<3> Ksh. 1000 - 5000*  *<4> Ksh. 5001 - 10,000*  *<5> Ksh. 10,001 - 15,000*  *<6> Ksh. 15,001 - 20,000*  *<7> Above Ksh. 20,001*  *<8> Don't know*  *<9> Can't remember* |
| *[Q4_12_3] Payment for legal services* | *<1> Not applicable*  *<2> Below Ksh. 1000*  *<3> Ksh. 1000 - 5000*  *<4> Ksh. 5001 - 10,000*  *<5> Ksh. 10,001 - 15,000*  *<6> Ksh. 15,001 - 20,000*  *<7> Above Ksh. 20,001*  *<8> Don't know*  *<9> Can't remember* |
| *[Q4_12_4] Payment for counselling* | *<1> Not applicable*  *<2> Below Ksh. 1000*  *<3> Ksh. 1000 - 5000*  *<4> Ksh. 5001 - 10,000*  *<5> Ksh. 10,001 - 15,000*  *<6> Ksh. 15,001 - 20,000*  *<7> Above Ksh. 20,001*  *<8> Don't know*  *<9> Can't remember* |
| *[Q4_12_5] Payment for accommodation* | *<1> Not applicable*  *<2> Below Ksh. 1000*  *<3> Ksh. 1000 - 5000*  *<4> Ksh. 5001 - 10,000*  *<5> Ksh. 10,001 - 15,000*  *<6> Ksh. 15,001 - 20,000*  *<7> Above Ksh. 20,001*  *<8> Don't know*  *<9> Can't remember* |
| *[Q4_12_6] Other Specify other expense in the space below* | *<1> Not applicable*  *<2> Below Ksh. 1000*  *<3> Ksh. 1000 - 5000*  *<4> Ksh. 5001 - 10,000*  *<5> Ksh. 10,001 - 15,000*  *<6> Ksh. 15,001 - 20,000*  *<7> Above Ksh. 20,001*  *<8> Don't know*  *<9> Can't remember* |
| Q4.13. Who paid for the services you received from the RRRP/GBVRC/shelter? | <1> Self  <2> Parents  <3> Blood relative  <4> Perpetrator  <5> Other member of marital family  <6> The RRRP/GBVRC/shelter  <7> Well-wisher  <8> Other (specify) |
| **SECTION 5: COPING AND RECOVERY DUE TO RRRP/GBVRC.** |  |
| 5.0. Respond to the following statements with regard to the help you received from GBVRC/RRRP. |  |
| Q5.1. The help made me to review my value in the society | <1> Yes  <2> No  <3> No response |
| Q5.2. The help prompted me to start standing up for myself by saying no to what I do not believe in | <1> Yes  <2> No  <3> No response |
| Q5.3. The help inspired me to join others campaigning against SGBV in my community or neighbourhood. | <1> Yes  <2> No  <3> No response |
| Q5.4a. The program enhanced my ability to participate in income generating activities | <1> Yes  <2> No  <3> No response |
| Q5.4b If yes, which income generating activities do you engage in? | <1> Selling grocery  <2> Hotel/restaurant  <3> Farming  <4> Dress making  <5> Baking  <6> Hair salon and beauty products  <7> Selling ready food on order  <8> Mobile hair and beauty services  <9> Sale of second hand clothes  <10> Other (specify) |
| Q5.5a. The help inspired me to join a women’s group | <1> Yes  <2> No  <3> No response |
| Q5.5b. If yes, what is the nature of the women’s group you joined? | <1> Investment group  <2> Table banking  <3> Therapy/counselling  <4> Community SGBV activist group  <5> Other (Specify) |
| Q5.6a. The help encouraged me to go for further education/training | <1> Yes  <2> No  <3> No response |
| Q5.6b If yes, what education/training did you pursue? | <1> Dressmaking  <2> Brickmaking  <3> Welding  <4> Hairdressing  <5> Electrical engineering  <6> Plumbing  <7> Teaching  <8> Preaching  <9> Other (Specify) |
| Q5.7. The help enabled me to seek employment | <1> Yes  <2> No  <3> No response |
| Q5.8a. The help motivated me to start saving | <1> Yes  <2> No  <3> No response |
| Q5.8b. If yes, which saving model do you use? | <1> Formal banking  <2> Mobile phone banking e.g. M-shwari  <3> Informal banking e.g. merry go rounds, women’s groups etc |
| Q5.8c. If yes, what is your level of savings per month? | <1> Not applicable  <2> Below Ksh. 1000  <3> Ksh. 1000 - 5000  <4> Ksh. 5001 - 10,000  <5> Ksh. 10,001 - 15,000  <6> Ksh. 15,001 - 20,000  <7> Above Ksh. 20,001  <8> Not willing to disclose |
| Q5.9a. The help enabled me to recover my lost property | <1> Yes  <2> No  <3> No response |
| Q5.9b. If yes, please explain |  |
| Q5.10a. The help enabled me to continue benefiting from family resources that were there before the violence. | <1> Yes  <2> No  <3> No response |
| Q5.10b. If yes, please explain |  |
| Q5.11a. The help enabled me start investing? | <1> Yes  <2> No  <3> No response |
| Q5.11b. If yes, please explain |  |
| Q5.12a. Based on the help you received from RRRP/GBVRC/shelter would you say that your life has changed for the better? | <1> Yes  <2> No  <3> No response |
| Q5.12b. Please explain |  |
| [Q6] What advice would you give to a woman going through SGBV? |  |
| [Q7] What would you recommend to the specific GBVRC/RRRP/shelter you benefited from on how to improve services for SGBV survivors? |  |
| [Q8] What would you recommend to the government on improving services to SGBV survivors? |  |
| [Q9]. Is there anything else you wish to add? | <1> Nothing |
| [GPS] Please capture GPS |  |
| [end_time] End time |  |
| [calc_time] Calculates time |  |
| [time_taken] Time taken: |  |
| End- Thank you very much for your time! Tap next to submit the survey! |  |
